# Identifying determinants of adherence to adjuvant endocrine therapy following breast cancer: A systematic review of reviews

**DOI:** 10.1002/cam4.6937

**Published:** 2024-01-19

**Authors:** Adam Todd, Catherine Waldron, Lucy McGeagh, Ruth Norris, Iakov Bolnykh, Sarah Jane Stewart, Joanna Slodkowska‐Barabasz, Zoe Moon, Caitriona Cahir, Sue Thompson, Victoria Harmer, Mary Wells, Eila Watson, Linda Sharp, the SWEET Research Team

**Affiliations:** ^1^ Newcastle University Newcastle upon Tyne UK; ^2^ Royal College of Surgeons in Ireland Dublin Ireland; ^3^ Oxford Brookes University Oxford UK; ^4^ The Newcastle upon Tyne Hospitals NHS Foundation Trust Newcastle upon Tyne UK; ^5^ University College London London UK; ^6^ Imperial College Healthcare NHS Trust London UK

**Keywords:** adherence, aromatase inhibitors, breast cancer, determinants, endocrine therapy, tamoxifen

## Abstract

**Background:**

In oestrogen‐receptor positive breast cancer, daily oral adjuvant endocrine therapy (ET) for at least 5 years significantly reduces risks of recurrence and breast cancer‐specific mortality. However, many women are poorly adherent to ET. Development of effective adherence support requires comprehensive understanding of influences on adherence. We undertook an umbrella review to identify determinants of ET adherence.

**Methods:**

We searched PubMed, Embase, CINAHL, PsycINFO, Cochrane and PROSPERO (inception to 08/2022) to identify systematic reviews on factors influencing ET adherence. Abstracted determinants were mapped to the World Health Organization's dimensions of adherence. Reviews were quality appraised and overlap assessed.

**Results:**

Of 5732 citations screened, 17 reviews were eligible (9 quantitative primary studies; 4 qualitative primary studies; 4 qualitative or quantitative studies) including 215 primary papers. All five WHO dimensions influenced ET non‐adherence: The most consistently identified non‐adherence determinants were patient‐related factors (e.g. lower perceived ET necessity, more treatment concerns, perceptions of ET ‘cons’ vs. ‘pros’). Healthcare system/healthcare professional‐related factors (e.g. perceived lower quality health professional interaction/relationship) were also important and, to a somewhat lesser extent, socio‐economic factors (e.g. lower levels of social/economic/material support). Evidence was more mixed for medication‐related and condition‐related factors, but several may be relevant (e.g. experiencing side‐effects, cost). Potentially modifiable factors are more influential than non‐modifiable/fixed factors (e.g. patient characteristics).

**Conclusions:**

The evidence‐base on ET adherence determinants is extensive. Future empirical studies should focus on less well‐researched areas and settings. The determinants themselves are numerous and complex in indicating that adherence support should be multifaceted, addressing multiple determinants.

## 1 INTRODUCTION

Breast cancer is the most commonly‐diagnosed cancer worldwide, with recent data suggesting there are over 2 million breast cancer cases diagnosed annually—equating to 11.7% of all cancer cases.^1^ Although survival outcomes for breast cancer continue to improve, each year there are still around 685,000 deaths globally.^2^ While there are different subtypes of breast cancer, the most common type (~75%) is oestrogen receptor (ER) positive—meaning the growth of the cancer is dependent upon the presence of oestrogen.^3^ Women with ER positive breast cancer are usually recommended to take oral adjuvant endocrine therapy (AET) for a number of years following surgery and/or radiotherapy. The use of AET has been shown to significantly improve outcomes for patients: the risk of disease recurrence, breast cancer‐related mortality and all‐cause mortality are reduced with use of AET.^4^, ^5^, ^6^ Indeed, extending the treatment duration of AET from 5 to 10 years, has been shown to further improve outcomes and reduce disease recurrence.^7^, ^8^ Examples of AET include the aromatase inhibitors (e.g. letrozole, anastrozole or exemestane), which are generally used for postmenopausal women, and tamoxifen which is generally used for pre‐menopausal women.

Despite the well‐recognised benefits of AET, there is evidence to suggest that people do not take it every day, while some people stop taking the AET altogether. Indeed, current estimations suggest that between 30 and 60% of women take less AET than recommended, while between 30 and 70% of women prematurely stop their AET at the end of 5 years treatment.^9^ There is strong evidence that not‐adhering to AET is associated with an increased risk of breast cancer reoccurrence and mortality.^10^, ^11^ Adherence is a complex behaviour influenced by many different factors. In response, the World Health Organization developed a model outlining five dimensions of medication adherence, which highlighted this issue is not exclusively a patient‐driven problem.^12^ Numerous studies have attempted to examine the broader factors why women do not adhere to AET. Indeed, several systematic reviews have been published on this topic that have synthesised and summarised this complex information. While systematic reviews are widely recognised as a robust way to appraise and synthesise available evidence, existing reviews on AET adherence are highly variable and have often focused exclusively on qualitative literature (e.g. Clancy et al.^13^) or quantitative literature (e.g. Cahir et al.^14^). Furthermore, some systematic reviews have focused on specific aspects of treatment adherence (e.g. only exploring adherence in the context of side effects from AET^15^), making it challenging to understand the broader factors underpinning AET adherence. A broad and comprehensive review identifying and summarising the different AET adherence determinants is needed to understand how best to support women to continue to take their AET, as well as identifying evidence gaps and informing future research endeavours. In this paper, we undertake a ‘review of reviews’ to examine different determinants of adherence to AET following breast cancer. A review of reviews is an established and effective way to bring together and summarise a broad evidence base, and this approach has been used to report a number of complex topics relating to adherence (e.g.^16^, ^17^).

## 2 METHODS

The review of reviews was registered with PROSPERO (CRD42020219950) and completed according to the PRISMA checklist (Supplementary File 1).

### 2.1 Inclusion criteria

Following standard evidence synthesis approaches, the inclusion criteria for the review of reviews were determined a priori in terms of PECOS (Population, Exposure, Comparison, Outcome and Study design).

Population: Women with breast cancer, in any country.

Exposure: AET (aromatase inhibitor or tamoxifen).

Comparison: No comparisons were required; comparisons could include adherence determinants to other breast cancer treatments, such as chemotherapy or targeted therapy.

Outcomes: Determinants of adherence to AET reported at the systematic review level (e.g. personal, clinical, economic, psychosocial). Adherence could be measured objectively (e.g. prescription encashment/refill data) or self‐reported through completion of questionnaires or lived experience.

Study design: Systematic reviews of (i) qualitative studies, (ii) quantitative studies or (iii) qualitative or quantitative studies. To meet the definition of a systematic review, four of the following five criteria had to be present:

- Inclusion/exclusion criteria were reported;
- The search strategy was reported;
- The included studies were synthesised;
- The quality of the included studies was assessed; and
- Sufficient details about the individual studies were presented.

### 2.2 Exclusion criteria

Systematic reviews investigating prevalence of (non‐)adherence to AET or exploring the effectiveness of interventions to promote adherence to AET were excluded. Systematic reviews published as conference abstracts only were also excluded, as were those published in languages other than English.

### 2.3 Search strategy

A search strategy was developed with support from an information specialist and executed in the following databases: PubMed, Embase, Cumulative Index to Nursing and Allied Health Literature (CINAHL), PsycINFO, Cochrane (Breast Cancer Group) and PROSPERO. The searches were carried out from database inception 3 August 2022. The search strategy is included in Supplementary File 2. Reference lists of included systematic reviews were further hand searched for relevant articles.

### 2.4 Study selection, and data extraction

Titles and abstracts were screened by a single reviewer (CW). The full text of all reviews identified for inclusion, together with any for which a decision on inclusion was not possible, were obtained for a more detailed examination. The full‐text articles were then assessed by one reviewer (CW or AT) and checked by another (ZM or JSB). Discrepancies at the full‐text screening stage were resolved through discussion. If agreement was not possible, a third reviewer was consulted (LS) who had consensus. If needed, authors of eligible reviews were contacted to confirm details relevant to eligibility; if no response was received within 2 weeks, a decision on inclusion was made based on the published information. Guided by the inclusion criteria, reasons for exclusion were recorded at the full text stage.

For each review, all data extraction was completed by one author (LMcG, LS, CC, SJS) and checked by a second (AT, EW, MW). Any disagreements were discussed with the wider group to reach consensus. Data extracted included bibliographic details and characteristics of the systematic review, methodology employed, primary studies included in each systematic review, synthesised determinants of adherence, conclusions. No data were abstracted from any of the original papers included in the eligible systematic reviews.

### 2.5 Synthesis

Narrative synthesis was undertaken at the systematic review level (i.e. the synthesis focussed on the reported findings, as stated in the eligible reviews; the original primary studies included in the reviews were not examined or extracted). Factors reported to be associated with adherence (reviews including quantitative studies) or themes/constructs reported to influence adherence (reviews including qualitative studies) were mapped to the World Health Organization's (WHO) five dimensions of adherence,^12^ and an updated conceptual model of these five dimensions.^18^ Each potential determinant was extracted and tabulated under the domains(s) it related to (namely: condition‐related factors; medication‐related factors; healthcare system/healthcare professional‐related factors; patient‐related factors; socio‐economic factors) and categorised as to whether the relevant systematic review stated that: it was seldom or never reported to influence adherence; the evidence for association was mixed; or it was associated with non‐adherence. To aid interpretation, and as our focus was on influences on *non‐adherence*, if a determinant was reported to be associated with (better/higher) adherence, we recorded the inverse as being associated with non‐adherence (e.g. if early‐stage disease was reported to be associated with better adherence, we recorded that later stage disease was associated with non‐adherence). We did not seek to distinguish between different types of non‐adherence (e.g. sub‐optimal implementation/early discontinuation) because the reviews did not report this.

### 2.6 Assessment of methodologic quality and study overlap

The quality of each systematic review was assessed by one author (LS, CC, SJS, LMcG) and checked by a second (EW, RN) using the JBI checklist for systematic reviews.^19^ Any disagreements were discussed with other AT who had consensus. Reviews were not excluded from the synthesis on the basis of low‐quality assessment. The overlap of primary studies was reported in a citation matrix and the corrected coverage area (CCA)^20^ was calculated for all reviews combined, and by study design(s) included in the reviews (i.e. quantitative only, qualitative only, qualitative or quantitative). The CCA is calculated using the following equation: $\mathrm{CCA}=N-r/\left(\mathrm{rc}-r\right)$, where *N* is the total number of primary publications from the included systematic reviews (including double counting); where *r* is the number of primary publications; and, *c* is the number of included systematic reviews.^20^ According the CCA, a value of 0–5 represents ‘slight’ overlap; 6–10 ‘moderate’ overlap; 11–15 ‘high’ overlap; and greater than 15, ‘very high’ overlap.

## 3 RESULTS

**FIGURE 1:**
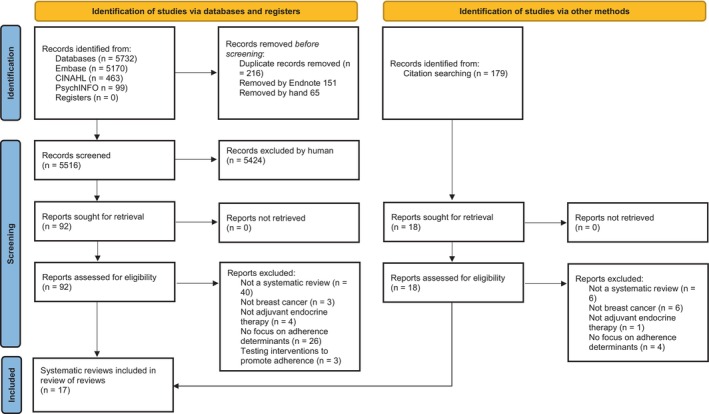
Study selection and exclusion according to the PRISMA statement.

After de‐duplication, searches provided 5732 records for screening; 92 full text papers were assessed. Screening reference lists of eligible reviews provided a further 179 citations of which 18 were reviewed in full text. The study selection process and reasons for exclusions are shown in Figure 1. Overall, 17 systematic reviews, reporting unique 215 primary studies, were eligible and included in the narrative synthesis.^9^, ^13^, ^14^, ^15^, ^21^, ^22^, ^23^, ^24^, ^25^, ^26^, ^27^, ^28^, ^29^, ^30^, ^31^, ^32^, ^33^

### 3.1 Characteristics of eligible reviews

Nine reviews, published between 2012 and 2022, included only quantitative primary studies^9^, ^14^, ^21^, ^22^, ^24^, ^26^, ^29^, ^30^, ^32^; these reviews included between 13 and 68 papers. Four reviews, published 2019–2021, included only qualitative primary studies^13^, ^15^, ^31^, ^33^ and included between 16 and 24 primary studies. Four reviews^23^, ^25^, ^27^, ^28^ included both qualitative and quantitative primary studies; they were published 2014–2019 and included 19–43 primary papers. The characteristics of the eligible reviews are summarised in Supplementary File 3.

All reviews aimed to synthesise determinants of adherence to AET, although several focussed on particular groups of determinants, such as adverse effects,^15^, ^22^ psychosocial factors^30^ or those which are potentially modifiable.^14^, ^29^ All reviews limited eligibility to primary studies published in English. One review limited consideration to studies of women with postmenopausal breast cancer^21^; 11 reviews focused only on women with breast cancer and excluded men (or populations who were predominately male)^9^, ^14^, ^15^, ^21^, ^22^, ^23^, ^24^, ^26^, ^29^, ^31^, ^32^; and some excluded studies of DCIS or people with Stage 4 cancer^14^, ^15^, ^22^ to maintain a focus on AET. All reviews undertook a narrative synthesis with three of the four qualitative reviews using a thematic synthesis approach^13^, ^15^, ^31^ and the fourth undertaking meta‐synthesis based on grounded theory.^33^ Two reviews conducted a meta‐analysis but could only do this for one^24^ or three^14^ potential modifiable adherence determinants. Nine reviews made some attempt to distinguish determinants of different types of non‐adherence (e.g. missing doses of AET or stopping AET earlier than planned), but the terms used were not consistent across the reviews.^9^, ^13^, ^14^, ^22^, ^24^, ^26^, ^27^, ^30^, ^33^ Of these nine reviews, one review of qualitative studies organised their findings in relation to different forms of non‐adherence, with different themes focusing on initiation, adherence and persistence,^33^ while a review of quantitative studies conducted meta‐analyses according to non‐adherence or non‐persistence.^13^

### 3.2 Review quality

Overall, the quality of the reviews was mixed (Supplementary File 4). Four^14^, ^15^, ^31^, ^32^ had ten ‘yes’ ratings out of a possible eleven; a further three^22^, ^23^, ^26^ had nine and three more^13^, ^29^, ^33^ had eight. One review^30^ had five ‘yes’ ratings, while one review^25^ had three ‘yes’ ratings. The reviews were most often rated ‘unclear’ in response to questions on whether: the likelihood of publication bias assessed; critical appraisal conducted by two or more reviewers independently; recommendations for policy and/or practice supported by the reported data.

### 3.3 Overlap between reviews

In total, the 17 systematic reviews included 215 unique primary papers in their syntheses. Almost half (*n* = 96; 46%) of primary studies were cited in only one review, 41 (19%) were cited in two, 28 were cited in 3 (13%) and the remaining 50 (23%) were cited in 4 or more reviews. Considering all included systematic reviews, the CCA was 0.09, representing slight overlap. For the reviews that included only quantitative primary papers, the CCA was 0.13; for the reviews that included both qualitative and quantitative, it was 0.12; and for the reviews that included only qualitative primary papers, it was 0.48; all of these are considered slight overlap (Supplementary File 4).

### 3.4 Condition‐related factors

Twelve reviews (eight of quantitative studies; four of quantitative or qualitative studies) reported on condition‐related factors and adherence^9^, ^21^, ^22^, ^23^, ^24^, ^25^, ^26^, ^27^, ^28^, ^29^, ^30^, ^32^ (Table 1). In terms of breast‐cancer related characteristics, including markers of disease severity (e.g. larger tumour size, lymph node involvement) and history of previous cancer‐directed treatment, the evidence regarding adherence was inconsistent: some reviews reported an association with non‐adherence,^21^, ^28^, ^32^ some reported mixed findings,^26^, ^32^ while others reported neutral findings.^26^ Regarding patient‐specific factors, reviews concluded that the presence of co‐morbidities was either associated with non‐adherence^21^, ^25^, ^28^, ^32^ or the findings were mixed.^26^, ^27^ Similarly, there was inconsistent evidence on the associations with mental health conditions, receipt of psychological help, and use of psychotropic medication and non‐adherence.^22^, ^23^, ^24^, ^27^, ^29^, ^30^, ^32^

**TABLE 1:** Condition‐related factors reported at the systematic review level presented according to their association with AET adherence.

Condition‐related factors^a^	Neutral or seldom	Mixed	Associated with non‐adherence
*Disease control*	‐	‐	‐
*Disease characteristics*			
Positive lymph nodes^b^	Moon		Banning
Secondary breast cancer			Banning
More advanced disease	Moon	Yussof	Sawesi
Tumour laterality	Moon		
Larger tumour size	Moon		
Previous radiotherapy	Moon		
No previous chemotherapy		Moon	Yussof
Previous surgery/mastectomy/breast conserving surgery	Moon		
*Patient specific*			
The presence of co‐morbidities		Paranjpe Moon	Banning Sawesi Montagna Yussof
Presence of depression/anxiety/distress (mental health conditions)		Toivonen Van Liew Fleming	Lambert Paranjpe Yussof Mausbach
Receipt of psychological help and use of psychotropic medication	Van Liew		
Later year at diagnosis		Murphy	
Lower global/cancer specific quality of life scores	Van Liew		
Menopausal status	Moon		

^a^ The factors presented in italics were taken directly from the conceptual model of adherence,^18^ which was used to guide the synthesis of the review of reviews.

^b^ The determinants presented under each factor are taken directly from the included systematic reviews and, as such, have a cancer specific context.

### 3.5 Medication‐related factors

Fourteen reviews (seven quantitative, three qualitative and four mixed) synthesised evidence on AET‐related factors (Table 2). In terms of medication regimen, five reviews concluded there was mixed evidence of an association between the patient experiencing polypharmacy and non‐adherence.^9^, ^14^, ^23^, ^26^, ^28^ Switching type of AET was linked with non‐adherence by three reviews^9^, ^27^, ^32^ but a fourth review judged the evidence as mixed.^26^ Two reviews reached different conclusions about non‐adherence and using tamoxifen versus aromatase inhibitors: Yussof et al. reported that using tamoxifen was associated with non‐adherence (compared with aromatase inhibitors),^32^ while Moon et al. reported the findings were mixed.^26^ Eight reviews (including two of qualitative evidence) reported that experiencing treatment‐ or disease‐related side‐effects influenced non‐adherence^14^, ^15^, ^23^, ^25^, ^27^, ^28^, ^32^, ^33^; three further reviews concluded evidence was mixed.^9^, ^22^, ^29^ Links between non‐adherence and lack of preparedness for side‐effects and poor side‐effect management of coping strategies were each reported in two reviews.^13^, ^15^, ^30^, ^33^ In terms of medication properties, four reviews observed that higher AET cost was related to non‐adherence^23^, ^27^, ^28^, ^33^; while another review concluded the evidence on this was mixed.^26^

**TABLE 2:** Medication‐related factors reported at the systematic review level presented according to their association with AET adherence.

Medication‐related factors^a^	Neutral or seldom	Mixed	Associated with non‐adherence
*Medication regimen*			
Polypharmacy^b^		Lambert Cahir Sawesi Murphy Moon	Montagna
No prior medication			Yussof
Long therapy duration			Sawesi
Switching endocrine therapy		Moon	Murphy Paranjpe Yussof
Using tamoxifen over AIs		Moon	Yussof
*Medication effects*			
Experiencing side effects (related to condition or disease)		Murphy Toivonen Fleming	Lambert Cahir Sawesi AlOmeir Peddie Paranjpe Montagna Yussof
Not linking side effects to AET		Toivonen	
Poor side effect management/coping			Clancy AlOmeir
Lack of preparedness for side effects			Peddie Van Liew
Use of alternative therapies			Moon
Using medications to manage AET side effects		Yussof	
*Medication properties*			
Higher cost of medication		Moon	Lambert Sawesi AlOmeir Paranjpe
Higher out of pocket costs		Murphy	Yussof

^a^ The factors presented in italics were taken directly from the conceptual model of adherence,^18^ which was used to guide the synthesis of the review of reviews.

^b^ The determinants presented under each factor are taken directly from the included systematic reviews and, as such, have a cancer specific context.

### 3.6 Healthcare system/healthcare professional‐related factors

Fourteen reviews (six quantitative, four qualitative, four quantitative or qualitative) reported on health professional or healthcare system factors^9^, ^13^, ^14^, ^15^, ^23^, ^25^, ^26^, ^27^, ^28^, ^29^, ^30^, ^31^, ^32^, ^33^ (Table 3). Nine of 10 reviews concluded that patient perception of lower quality interactions, relationships or communication with health professionals was related to non‐adherence^13^, ^15^, ^23^, ^25^, ^26^, ^27^, ^28^, ^30^, ^31^ All eight reviews which commented on support from health professionals observed that lack of support was associated with non‐adherence.^13^, ^15^, ^23^, ^26^, ^30^, ^31^, ^32^, ^33^ Non‐adherence was also judged to be associated with: insufficient information provided from health professionals, or information that patients found hard to understand^13^, ^15^, ^23^, ^26^, ^30^, ^31^, ^33^; not valuing or trusting the health professionals' opinions^26^, ^29^, ^33^; and lack of involvement of a breast cancer specialist in follow‐up care.^14^, ^15^, ^23^, ^26^, ^32^, ^33^ Fewer reviews commented on health system‐related factors: two concluded that irregular or inconsistent follow‐up care was associated with non‐adherence^23^, ^32^ and two reported more hospitalisations were linked to non‐adherence.^26^, ^32^

**TABLE 3:** Healthcare system/healthcare professional‐related factors reported at the systematic review level presented according to their association with AET adherence.

Healthcare system/HCP‐related factors^a^	Neutral or seldom	Mixed	Associated with non‐adherence
*HCP characteristics*			
Perceived lower quality of HCP interaction/communication/relationships^b^		Toivonen	Lambert Sawesi Clancy Peddie Xu Paranjpe Van Liew Moon Montagna
Lack of HCP support			Lambert Clancy AlOmeir Peddie Xu Van Liew Moon Yussof
Insufficient/difficult to understand HCP information		Toivonen	Lambert Clancy AlOmeir Peddie Xu Van Liew Moon
Lack of involvement in breast cancer specialist in follow up care		Murphy	Lambert Cahir AlOmeir Peddie Moon Yussof
Not valuing/trusting HCP opinion			Toivonen AlOmeir Moon
Lack of discussion outlining the need for treatment	Van Liew		
Less frequent physician communication			Moon
The lack of strength in recommending AET			Toivonen Clancy
*Healthcare system‐related factors*			
Lack of a referral to an oncologist		Murphy	
Lack of opportunity for shared decision making		Toivonen Van Liew Moon	Montagna
Irregular or lack of continuity in follow up care			Lambert Yussof
Lack of transport			Paranjpe
More hospitalisations			Moon Yussof
*Other*			
Being involved in a research study			Lambert

^a^ The factors presented in italics were taken directly from the conceptual model of adherence,^18^ which was used to guide the synthesis of the review of reviews.

^b^ The determinants presented under each factor are taken directly from the included systematic reviews and, as such, have a cancer specific context.

### 3.7 Patient‐related factors

Reviews included a wide range of patient‐related factors; these were reported in 14 reviews (five quantitative, four qualitative, five quantitative or qualitative)^9^, ^13^, ^15^, ^21^, ^23^, ^25^, ^26^, ^27^, ^28^, ^29^, ^30^, ^31^, ^32^, ^33^ (Table 4). Non‐adherence was reported to be influenced by a variety of cognitive and psychological factors including: a perceived lack of benefit of AET^15^, ^21^, ^25^, ^32^; lower perceived necessity of treatment^27^, ^30^, ^31^, ^32^, ^33^; negative beliefs or concerns about AET^23^, ^26^, ^27^, ^28^; and negative emotional or attitudes towards AET.^25^, ^26^, ^27^ Lack of knowledge or understanding about breast cancer or AET was linked with non‐adherence in four reviews,^23^, ^28^, ^31^, ^33^ and belief that missing doses will not impact efficacy in three reviews.^23^, ^31^, ^33^ Not fearing cancer recurrence, or perceiving self as at low risk of recurrence was judged to be associated to non‐adherence in five reviews^13^, ^15^, ^25^, ^27^, ^30^, ^33^; two further reviews considered evidence on this mixed.^26^, ^29^

**TABLE 4:** Patient‐related factors reported at the systematic review level presented according to their association with AET adherence.

Patient‐related factors^a^	Neutral or seldom	Mixed	Associated with non‐adherence
*Cognitive and psychological factors*			
Perceived lack of benefit^b^			Banning Peddie Montagna Yussof
Lower perceived necessity of AET		Toivonen Moon	AlOmeir Xu Paranjpe Van Liew Yussof
Negative health beliefs			Banning
Negative beliefs/concerns about AET	Toivonen		Lambert Sawesi Paranjpe Moon
Internal locus of control	Toivonen		
Emotional representation	Toivonen		
Being able to cope	Toivonen		
Perceived sensitivity to medicine	Toivonen		
General distrust in medication			Lambert Montagna
Belief that missing doses won't impact efficacy			Lambert AlOmeir Xu
Lack of understanding/knowledge about AET/breast cancer			Lambert Sawesi AlOmeir Xu
Positive emotions/attitude to AET		Toivonen	
Negative emotions/attitude to AET		Toivonen	Paranjpe Moon Montagna
Not fearing cancer occurrence/perceiving as low risk		Toivonen Moon	Clancy AlOmeir Peddie Paranjpe Van Liew Montagna
Perceived lack of control over treatment		Toivonen	
Lack of coherence		Toivonen	Moon
Low protection/motivation			Toivonen
Lack of intention			Xu
Lack of optimism			Toivonen Moon
*Behavioural factors*			
Lower self‐efficacy* *take the medication; physician communication		Toivonen	Lambert Xu Paranjpe Van Liew Moon Montagna
Forgetfulness			Banning Lambert Sawesi AlOmeir
Poor medication taking routines			Lambert Paranjpe
Not intending to take AET		Toivonen	
Consulting with HCP when having trouble	Toivonen		
Practical problems associated with medication taking			Moon
*Priorities*			
Negative decisional balance (i.e. weight of pros versus cons)		Toivonen	Lambert Clancy AlOmeir Peddie Xu Van Liew Moon Montagna
Moving on from breast cancer experience/getting back to normal/focusing on the ‘now’, rather than future risk of occurrence/discounting future benefits			Lambert Xu Montagna
Fertility preservation			Lambert Moon
Therapy interfering with lifestyle			Sawesi
*Non‐modifiable characteristics*			
Younger age		Murphy Moon	Banning Sawesi Paranjpe Yussof
Older age		Murphy Moon	Sawesi Yussof
Ethnic minority		Moon Yussof	Sawesi
Personality characteristics			Lambert
Higher CYP2D6 activity			Murphy Yussof
*Family/caregiver characteristics*			
Having a young family			Lambert
Being unmarried			Sawesi Paranjpe Yussof
*Other*			
Lower health score			Banning

^a^ The factors presented in italics were taken directly from the conceptual model of adherence,^18^ which was used to guide the synthesis of the review of reviews.

^b^ The determinants presented under each factor are taken directly from the included systematic reviews and, as such, have a cancer specific context.

In terms of behavioural factors, six of seven reviews concluded that lower self‐efficacy influenced non‐adherence.^23^, ^25^, ^26^, ^27^, ^29^, ^30^, ^31^ Non‐adherence was linked to forgetfulness in four reviews,^21^, ^23^, ^30^, ^33^ poor medication‐taking routines in two reviews,^23^, ^27^ and practical problems with medication‐taking in one review.^26^

As regards priorities, eight of nine reviews reported that non‐adherence was associated with negative decisional balance (i.e. cons for taking AET outweighing pros).^13^, ^15^, ^23^, ^25^, ^26^, ^30^, ^31^, ^33^ Three concluded that a desire to ‘move on’ from breast cancer, ‘get back to normal’ and/or discounting future benefits influenced non‐adherence^23^, ^25^, ^31^; two stated that fertility preservation influenced non‐adherence^23^, ^26^; and one judged that the treatment interfering with lifestyle was important.^28^

In terms of personal and family/caregiver characteristics, younger age was linked to non‐adherence in four of six reviews,^21^, ^27^, ^28^, ^32^ as was older age in two of four reviews.^28^, ^32^ Being unmarried was reported to influence non‐adherence in three reviews.^27^, ^28^, ^32^

### 3.8 Socio‐economic factors

Evidence on socio‐economic factors influencing adherence was reported in 11 reviews (four quantitative, three qualitative, four quantitative or qualitative)^13^, ^15^, ^23^, ^25^, ^26^, ^27^, ^28^, ^29^, ^30^, ^32^, ^33^ (Table 5). The most extensively evaluated socio‐environmental factor was lack of social, emotional or material support, which seven of eight reviews concluded there was associations with non‐adherence.^23^, ^25^, ^26^, ^27^, ^30^, ^32^, ^33^ Single reviews reported links between non‐adherence and: having a family member with breast cancer^23^; lack of partner support^13^; and not feeling an obligation to family members.^15^ One review commented on lifestyle factors, observing that smoking was related to non‐adherence.^28^ In terms of economic factors, three reviews stated that there were relationships between lack of health insurance and non‐adherence,^23^, ^27^, ^33^ while single reviews reported links between non‐adherence and higher education,^27^ being in paid employment,^27^ working in a medical‐related profession,^23^ having a burdensome work schedule^28^ and lower financial status.^32^

**TABLE 5:** Socioeconomic factors reported at the systematic review level presented according to their association with AET adherence.

Socioeconomic factors^a^	Neutral or seldom	Mixed	Associated with non‐adherence
*Social/environmental factors*			
Having a family member appear with breast cancer^b^			Lambert
Engaging in religious practices	Toivonen		Sawesi
Lower levels of social/emotional/material/support		Toivonen	Lambert AlOmeir Paranjpe Van Liew Moon Montagna Yussof
Lower level of instrumental support	Van Liew		
Lack of partner support			Clancy
Not feeling an obligation or duty to family members			Peddie
Lack of informal support (variety of things such as internet).			Clancy
*Lifestyle factors*			
Smoking			Sawesi
*Economic factors*			
Lack of health insurance			Lambert AlOmeir Paranjpe
Higher education			Paranjpe
Being in paid employment			Paranjpe
Working in a medical related profession			Lambert
Burdensome work schedule			Sawesi
Lower financial status			Yussof

^a^ The factors presented in italics were taken directly from the conceptual model of adherence,^18^ which was used to guide the synthesis of the review of reviews.

^b^ The determinants presented under each factor are taken directly from the included systematic reviews and, as such, have a cancer specific context.

## 4 DISCUSSION

The high incidence of ER + ve breast cancer coupled with the high prevalence of non‐adherence (both suboptimal implementation and early discontinuation) means adherence to AET is an important public health problem. This umbrella review identifying determinants of adherence to AET included 17 systematic reviews and showed evidence for factors within all five of the WHO dimensions of medication adherence. The most consistently identified determinants were patient‐related factors (e.g. cognitive and psychological, such as having a lower perceived necessity for AET). Healthcare system/healthcare professional‐related factors (e.g. healthcare professional characteristics, such as having a perceived lower quality interaction or relationship with a healthcare professional) were also important and, to a somewhat lesser extent, socio‐economic factors (e.g. social/environmental such as a patient having lower levels of social support). Evidence was more mixed as regards medication‐related and condition‐related factors.

The primary conclusion from our synthesis is that non‐adherence to AET in women with breast cancer is complex and affected by multiple determinants. This echoes conclusions from umbrella reviews on adherence to medications for cardiovascular conditions and diabetes, and for medications more generally.^17^, ^34^, ^35^ Modifiable factors appear to be much more important than non‐modifiable factors. The former include some that are ‘inherent’ to the individual (such as how they weigh the pros and cons of taking the medication, or their self‐efficacy to take it), and external factors including both the individual's ‘personal’ context (such as social, emotional or material support) and the wider healthcare/health system context (such as perceived quality of communication and relationships with healthcare professionals or lack of involvement of breast cancer specialists in the individual's follow‐up). The implications of this are two‐fold: first, that predicting whether any specific patient prescribed AET will face problems with adherence based on their sociodemographic characteristics will be challenging and, second, that adherence support needs to be multifaceted.

It is clear that there is a significant body of literature on influences on adherence to endocrine therapy. The 17 included systematic reviews included 215 unique primary papers. In addition to these systematic reviews, there are several other reviews which did not meet our rigorous definition for a systematic review (see, e.g. Moore,^36^ Hadji^37^). This volume of primary and secondary data raises the question of whether more primary research is needed on this topic. However, it is worth noting that much of the evidence‐base accrues from the USA where the complexity of the healthcare system may drive some of the findings of the systematic reviews and hence of our synthesis. An example is the higher cost of the medication which several reviews concluded was associated with non‐adherence^23^, ^27^, ^28^, ^33^—this may be less of an issue in a universal healthcare system. Further research on determinants is most warranted in healthcare settings or certain clinical contexts that have been less extensively investigated; for example, low and middle income countries or younger people using AET who wish to become pregnant. Of note, the POSITIVE (Pregnancy Outcome and Safety of Interrupting Therapy for Women with Endocrine Responsive Breast Cancer) clinical trial seeks to address the question if AET can be paused, while a person aims to get pregnant.^38^

Our synthesis also showed that some categories of potential adherence determinants have not yet been fully investigated. For example, tamoxifen is metabolised by various cytochrome P450, UDP‐glucuronosyltransferase and sulfotransferase enzymes; all enzymes in the pathway are encoded by polymorphic genes.^39^ Enzymes in these and other pathways also metabolise aromatase inhibitors.^40^ Only two systematic reviews mentioned genetic factors in adherence, concluding that higher CYP2D6 activity was associated with non‐adherence.^9^, ^32^ This reflects the paucity of primary research on this topic. Better understanding of the role of these factors in influencing adherence—either directly or indirectly via other determinants (e.g. treatment adverse‐effects)—would be worthwhile. Lifestyle factors were mentioned in one review, which concluded that smoking was associated with non‐adherence.^28^ Smoking is associated with worse breast cancer survival^41^ and the finding regarding adherence suggests an explanation for that association. Various other lifestyle factors (including body fat and physical inactivity)^42^ have been associated with breast cancer survival and understanding whether those are also related to (non‐)adherence would be of value.

Although individual reviews were generally appraised as being of reasonable quality, it was striking how little overlap there was in the primary studies that they included. This is demonstrated in the overall CCA of 0.09 and the fact that almost half of the primary studies were included in only one systematic review. While some systematic reviews had a more specific or narrow focus (e.g. treatment adverse‐effects, or psychosocial factors) the lack of overlap was surprising. It may be that primary papers on this topic are difficult to identify systematically; a variety of terms for ‘non‐adherence’ are used in studies and reviews and the studies themselves may be published in journals with a diverse range of disciplines. To advance understanding in this area, and to aid future syntheses of findings, it vital that future primary studies, and systematic reviews, adopt standardised terminology (e.g. the European consensus on taxonomy and terminology of adherence).^43^

It was striking that only two of the reviews which included quantitative studies undertook meta‐analysis.^14^, ^24^ A small number of reviews were reasonably explicit about how they had assessed the primary evidence (e.g. Moon et al.^26^), but most simply provided a narrative synthesis of studies in which it was unclear how they had judged whether there was ‘sufficient’ evidence (e.g. measured in terms of number of studies, consistency of findings) to conclude any particular factor was associated with (non)adherence. This should be borne in mind when interpreting our synthesis. A further issue to consider is that it is highly likely that the ‘same’ determinant was assessed or interpreted in different ways in the primary studies included in the eligible reviews; we did not assess this as our synthesis was at the systematic review level. Thus, we concur with the comments from Leslie et al.^34^ on adherence to cardiovascular medicines—that (AET) adherence research is highly heterogeneous and efforts to standardise it are needed to improve comparability and facilitate future efforts to more precisely synthesise findings.

### 4.1 Implications

Increasing the effectiveness of adherence interventions may have a far greater impact on the health of the population than any improvement in specific medical treatments.^44^ Despite clinical trial data highlighting—two decades ago—that non‐adherence of AET was a significant problem,^45^, ^46^ how best to support women to adhere remains unclear. Guidance for development of complex interventions notes the importance of having a comprehensive understanding of determinants of the behaviour.^47^ We undertook this umbrella review to bring together the evidence on determinants to inform development of a complex intervention to support adherence to AET (see^48^ for more information).

Early interventions in this area were largely based on ‘education’ and were ineffective.^49^ While four of the eligible reviews found that lack of understanding or knowledge about breast cancer or AET was associated with non‐adherence,^23^, ^28^, ^31^, ^33^ it is generally accepted that improving knowledge is insufficient on its own to influence behaviour.^50^ Rather, as suggested by our synthesis, any intervention will need to be multifaceted seeking to address a wide variety of determinants. Our synthesis suggests that an intervention will need to pay particular attention to patient‐related cognitive and psychological factors, including women's perceptions of their need to take the therapy (including their perceptions of their recurrence risk) and the potential benefits of doing so, as well as any negative beliefs or concerns. These factors should be acknowledged and addressed by any healthcare professional with responsibility for initiating and prescribing AET. Building self‐efficacy while also addressing negative decisional balance (i.e. how women weigh the pros and cons of the therapy), and finding ways to positively frame adherence for women whose primary desire is to ‘move on’ from breast cancer will also be important. Other elements might include specific strategies to address forgetfulness and non‐intentional adherence. Given the importance of the healthcare context and some social/environmental factors, an intervention may also need to include elements that would lead to improved patient‐healthcare professional communication or relationships and more (or better quality) social and/or emotional support.

### 4.2 Strengths & limitations

We followed systematic review methods, searching multiple databases and applying clear criteria for what we considered a systematic review. Nonetheless, there are some limitations. We did not independently double screen all citations, but at least two authors assessed each paper at the full text stage. We did not search databases of PhD theses or conduct forward citation searching from the eligible reviews. Moreover, we excluded reviews not published in English (this restriction was also applied in all of the eligible systematic reviews). It is therefore possible that we may have missed eligible reviews. We also acknowledge that the quality of included reviews was mixed.

We chose to use the term ‘(non‐)adherence’ in our synthesis to capture all the different ways patients may fail to take AET as prescribed and because most of the eligible systematic reviews lacked a clear definition of adherence. As others have noted, research on adherence to AET—and medications in general—is bedevilled by inconsistent definitions and terminology.^17^ We were therefore unable to evaluate whether the determinants of different types of non‐adherence (i.e. non‐initiation, sub‐optimal implementation and early discontinuation) differ. Some data indicate that women who have suboptimal implementation of AET are at increased risk of future early discontinuation^51^ suggesting that the primary focus of adherence interventions should be to prevent suboptimal implementation. However, from the published data included in our synthesis is it impossible to clearly conclude which of the array of determinants identified here should be targeted to prevent this as opposed to early discontinuation.

While the WHO's five dimensions of adherence offer a useful approach to summarise correlates of non‐adherence to date, the utility of this framework as an approach to guide intervention development has limitations^52^ largely because the dimensions do not provide any understanding of the mechanisms through which each factor acts on adherence. Future work could therefore consider the review findings alongside the context of theoretical models, such as the Perceptions and Practicalities Approach (PaPA),^53^ which help explain how these factors act on adherence through either a patients' motivation or ability.

### 4.3 Conclusion

The evidence‐base exploring and investigating AET determinants—at the condition‐related, medication‐related, healthcare system/healthcare professional‐related, patient‐related, socioeconomic‐related levels—is extensive. Future research should focus on gaps and inconsistencies in this evidence base (e.g. economic and lifestyle factors), rather than repeat what is already known and well established. In terms of implications, adherence to AET is multifactorial and adherence support should acknowledge and address this complexity.

## AUTHOR CONTRIBUTIONS

**Adam Todd:** Funding acquisition (equal); investigation (equal); methodology (equal); supervision (equal); writing – original draft (equal). **Catherine Waldron:** Investigation (equal); writing – review and editing (equal). **Lucy McGeagh:** Investigation (equal); methodology (equal); writing – review and editing (equal). **Ruth Norris:** Investigation (equal); writing – review and editing (equal). **Iakov Bolnykh:** Investigation (equal); writing – review and editing (equal). **Sarah Jane Stewart:** Investigation (equal); writing – review and editing (equal). **Joanna Slodkowska‐Barabasz:** Investigation (equal); writing – review and editing (equal). **Zoe Moon:** Investigation (equal); writing – review and editing (equal). **Caitriona Cahir:** Funding acquisition (equal); investigation (equal); methodology (equal); writing – review and editing (equal). **Sue Thompson:** Investigation (equal); project administration (equal); writing – review and editing (equal). **Victoria Harmer:** Writing – review and editing (equal). **Mary Wells:** Funding acquisition (equal); investigation (equal); methodology (equal); writing – review and editing (equal). **Eila Watson:** Funding acquisition (equal); investigation (equal); methodology (equal); writing – review and editing (equal). **Linda Sharp:** Funding acquisition (equal); investigation (equal); methodology (equal); writing – original draft (equal).

## FUNDING INFORMATION

This article presents independent research funded by the National Institute for Health and Care Research (NIHR) under the Programme Grants for Applied Research programme [NIHR200098]. The views expressed in this article are those of the authors and not necessarily those of the NHS, the NIHR or the Department of Health. The funder had no role in: the study design or conduct or interpretation of the data; writing the manuscript; or the decision to submit the article for publication. AT and LS are part funded by the National Institute for Health and Care Research (NIHR) Newcastle Patient Safety Research Collaboration (PSRC). The views expressed are those of the author(s) and not necessarily those of the NIHR or the Department of Health and Social Care.

## CONFLICT OF INTEREST STATEMENT

Linda Sharp, Eila Watson, Adam Todd, Mary Wells, Caitriona Cahir reports financial support was provided by National Institute of Health and Care Research.

## ETHICS STATEMENT

Not applicable as the presented work is evidence synthesis.

## PATIENT CONSENT STATEMENT

Not applicable as the presented work is evidence synthesis.

## Supporting information


Supplementary File 1.

Supplementary File 2.

Supplementary File 3.

Supplementary File 4.

Supplementary File 5.

## Data Availability

The datasets generated and/or analysed during the current study are included within the article and the supplementary information.
