# Supplementary material for: Identifying determinants of adherence to adjuvant endocrine therapy following breast cancer: A systematic review of reviews
Source: Cancer Med. 2024 Jan 19;13(3):e6937. doi: 10.1002/cam4.6937 (PMC10905548; doi:10.1002/cam4.6937)
Supplement: Supplementary file 2 [file CAM4-13-e6937-s001.docx]

**Supplementary File 1**

**Embase**

**#1** ('breast'/exp OR breast) AND carcinoma* OR (('breast'/exp OR breast) AND ('cancer'/exp OR cancer)) OR (('breast'/exp OR breast) AND malignan*) OR (('breast'/exp OR breast) AND tumo?r*) OR (('breast'/exp OR breast) AND neoplasm*) OR 'breast cancer'/exp OR 'breast cancer'

**AND #2** ((((((((('adjuvant'/exp OR 'adjuvant' OR 'adjuvant'/exp OR adjuvant) AND endocrine AND therap* OR 'adjuvant'/exp OR 'adjuvant' OR 'adjuvant'/exp OR adjuvant) AND hormonal AND therap* OR 'aromatase'/exp OR 'aromatase' OR 'aromatase'/exp OR aromatase) AND inhibitor* OR antineoplastic) AND agents, AND hormonal+ OR 'estrogen'/exp OR 'estrogen' OR 'estrogen'/exp OR estrogen) AND ('receptor'/exp OR 'receptor' OR 'receptor'/exp OR receptor) AND modulators+ OR 'estrogen'/exp OR 'estrogen' OR 'estrogen'/exp OR estrogen) AND antagonists+ OR 'adjuvant'/exp OR 'adjuvant' OR 'adjuvant'/exp OR adjuvant) AND endocrine AND therap* OR 'adjuvant'/exp OR 'adjuvant' OR 'adjuvant'/exp OR adjuvant) AND hormonal AND therap* OR 'aromatase'/exp OR 'aromatase' OR 'aromatase'/exp OR aromatase) AND inhibitor* OR 'tamoxifen'/exp OR 'tamoxifen' OR 'tamoxifen'/exp OR tamoxifen OR 'anastrozole'/exp OR 'anastrozole' OR 'anastrozole'/exp OR anastrozole OR 'letrozole'/exp OR 'letrozole' OR 'letrozole'/exp OR letrozole OR 'fulvestrant'/exp OR 'fulvestrant' OR 'fulvestrant'/exp OR fulvestrant OR 'exemestane'/exp OR 'exemestane' OR 'exemestane'/exp OR exemestane OR 'toremifene'/exp OR 'toremifene' OR 'toremifene'/exp OR toremifene OR 'raloxifene'/exp OR 'raloxifene' OR 'raloxifene'/exp OR raloxifene

**AND** **#3 OR #4**

**#3** (((((((((('adherence'/exp OR 'adherence' OR 'adherence'/exp OR adherence OR non) AND ('adherence'/exp OR 'adherence' OR 'adherence'/exp OR adherence) OR nonadherence OR 'compliance'/exp OR 'compliance' OR 'compliance'/exp OR compliance OR non) AND ('compliance'/exp OR 'compliance' OR 'compliance'/exp OR compliance) OR noncompliance) AND n3 AND ('treatment'/exp OR 'treatment' OR 'treatment'/exp OR treatment OR 'medication'/exp OR 'medication' OR 'medication'/exp OR medication OR patient*) OR persist* OR discontinue*) AND n3 AND ('treatment'/exp OR 'treatment' OR 'treatment'/exp OR treatment OR 'medication'/exp OR 'medication' OR 'medication'/exp OR medication OR patient*) OR (('treatment'/exp OR 'treatment' OR 'treatment'/exp OR treatment) AND ('medication'/exp OR 'medication' OR 'medication'/exp OR medication) AND n2 AND refus*) OR 'treatment'/exp OR 'treatment' OR 'treatment'/exp OR treatment) AND ('compliance'/exp OR 'compliance' OR 'compliance'/exp OR compliance) OR 'treatment'/exp OR 'treatment' OR 'treatment'/exp OR treatment) AND ('refusal'/exp OR 'refusal' OR 'refusal'/exp OR refusal) OR 'treatment'/exp OR 'treatment' OR 'treatment'/exp OR treatment) AND ('dropouts'/exp OR 'dropouts' OR 'dropouts'/exp OR dropouts) OR 'patient'/exp OR 'patient' OR 'patient'/exp OR patient) AND ('compliance'/exp OR 'compliance' OR 'compliance'/exp OR compliance) OR 'medication'/exp OR 'medication' OR 'medication'/exp OR medication) AND ('compliance'/exp OR 'compliance' OR 'compliance'/exp OR compliance) OR 'treatment'/exp OR 'treatment' OR 'treatment'/exp OR treatment) AND ('refusal'/exp OR 'refusal' OR 'refusal'/exp OR refusal) OR 'implementation'/exp OR 'implementation' OR 'implementation'/exp OR implementation OR discontinuation OR 'persistence'/exp OR 'persistence' OR 'persistence'/exp OR persistence OR 'initiation'/exp OR 'initiation' OR 'initiation'/exp OR initiation OR pharmonics OR ((compliance OR 'non compliance' OR noncompliance OR adherence OR 'non adherence' OR nonadherence OR persist* OR discontinue*) NEAR/3 (treatment OR medication OR patient*)) OR 'treatment refusal'/exp OR 'treatment refusal' OR 'patient compliance'/exp OR 'patient compliance'

**#4** (((((((((((((((((((((((('social'/exp OR 'social' OR 'social'/exp OR social) AND ('support'/exp OR 'support' OR 'support'/exp OR support) OR 'social'/exp OR 'social' OR 'social'/exp OR social) AND networks+ OR 'social'/exp OR 'social' OR 'social'/exp OR social) AND groups OR 'social'/exp OR 'social' OR 'social'/exp OR social) AND ('determinants'/exp OR 'determinants' OR 'determinants'/exp OR determinants) AND of AND ('health'/exp OR 'health' OR 'health'/exp OR health) OR 'social'/exp OR 'social' OR 'social'/exp OR social) AND disadvantage OR 'family'/exp OR 'family' OR 'family'/exp OR family) AND ('support'/exp OR 'support' OR 'support'/exp OR support) OR 'family'/exp OR 'family' OR 'family'/exp OR family) AND ('caregivers'/exp OR 'caregivers' OR 'caregivers'/exp OR caregivers) OR 'social'/exp OR 'social' OR 'social'/exp OR social) AND ('support'/exp OR 'support' OR 'support'/exp OR support) OR 'social'/exp OR 'social' OR 'social'/exp OR social) AND ('stigma'/exp OR 'stigma' OR 'stigma'/exp OR stigma) OR 'employment'/exp OR 'employment' OR 'employment'/exp OR employment) AND status OR 'rural'/exp OR 'rural' OR 'rural'/exp OR rural OR lack) AND of AND provider AND ('caregiver'/exp OR 'caregiver' OR 'caregiver'/exp OR caregiver) AND ('availability'/exp OR 'availability' OR 'availability'/exp OR availability) OR poor) AND ('access'/exp OR 'access' OR 'access'/exp OR access) AND to AND ('healthcare'/exp OR 'healthcare' OR 'healthcare'/exp OR healthcare) OR follow) AND up OR presence) AND of AND ('symptoms'/exp OR 'symptoms' OR 'symptoms'/exp OR symptoms) OR 'disease'/exp OR 'disease' OR 'disease'/exp OR disease) AND ('severity'/exp OR 'severity' OR 'severity'/exp OR severity) OR 'clinical'/exp OR 'clinical' OR 'clinical'/exp OR clinical) AND improvement OR psychiatric) AND ('condition'/exp OR 'condition' OR 'condition'/exp OR condition) OR 'duration'/exp OR 'duration' OR 'duration'/exp OR duration) AND of AND the AND ('disease'/exp OR 'disease' OR 'disease'/exp OR disease) OR adverse) AND effects OR 'patient'/exp OR 'patient' OR 'patient'/exp OR patient) AND ('friendliness'/exp OR 'friendliness' OR 'friendliness'/exp OR friendliness) AND of AND the AND regimen OR perceived) AND effectiveness AND of AND the AND ('drug'/exp OR 'drug' OR 'drug'/exp OR drug) OR 'duration'/exp OR 'duration' OR 'duration'/exp OR duration) AND of AND the AND ('treatment'/exp OR 'treatment' OR 'treatment'/exp OR treatment) OR 'drug'/exp OR 'drug' OR 'drug'/exp OR drug) AND type OR 'health'/exp OR 'health' OR 'health'/exp OR health) AND ('beliefs'/exp OR 'beliefs' OR 'beliefs'/exp OR beliefs) OR 'knowledge'/exp OR 'knowledge' OR 'knowledge'/exp OR knowledge OR 'co morbidities' OR 'social determinants of health'/exp OR 'social determinants of health' OR 'caregiver support'/exp OR 'caregiver support' OR 'social support'/exp OR 'social support' OR 'employment status'/exp OR 'employment status' OR 'health care access'/exp OR 'health care access' OR 'adverse drug reaction'/exp OR 'adverse drug reaction'

**AND [review]/lim AND [english]/lim AND [abstracts]/lim**

**CINAHL**

SU Breast Neoplasms OR ( breast n3 (cancer OR neoplasm* OR tumo?r* OR carcinoma)) OR (AB breast n3 (cancer OR neoplasm* OR tumo?r* OR carcinoma) )

**AND** ( adjuvant endocrine therap* OR adjuvant hormonal therap* OR aromatase inhibitor*) ) OR MH Antineoplastic Agents, Hormonal+ OR MH Estrogen Receptor Modulators+ OR MH Estrogen Antagonists+’ OR ( adjuvant endocrine therap* OR adjuvant hormonal therap* OR aromatase inhibitor* ) OR ( tamoxifen OR anastrozole OR letrozole OR fulvestrant OR exemestane OR toremifene OR raloxifene )

**AND** ( ( (adherence OR non adherence OR nonadherence OR compliance OR non compliance OR noncompliance) N3 (treatment OR medication OR patient*) ) OR AB ( (adherence OR non adherence OR nonadherence OR compliance OR non compliance OR noncompliance) N3 (treatment OR medication OR patient*) ) OR AB ((persist* OR discontinu*) N3 (treatment OR medication OR patient*) ) OR TI ((persist* OR discontinu*) N3 (treatment OR medication OR patient*) ) ) OR ( (Treatment OR medication) N2 refus* ) OR AB ( (Treatment OR medication) N2 refus* ) ) OR ( patient compliance or concordance or adherence ) OR MH patient compliance+ OR ( medication compliance or medication adherence or medication non-compliance or medication non-adherence ) OR patient dropouts OR treatment refusal OR ( (implementation OR discontinuation OR persistence OR initiation OR pharmionics) OR AB (implementation OR discontinuation OR persistence OR initiation OR pharmionics) ) OR (Family support OR Family caregivers OR Social support OR Social stigma OR Employment status OR Rural OR Lack of provider caregiver availability OR Poor access to healthcare OR Follow up OR Presence of symptoms OR Disease severity OR Clinical improvement OR Psychiatric condition OR Duration of the disease OR Adverse effects OR Patient friendliness of the regimen OR Perceived effectiveness of the drug OR Duration of the treatment OR Drug type OR Health beliefs OR Knowledge OR Co-morbidities)

**AND** Review

**Limiters - Abstract Available, Expanders - Apply equivalent subjects Narrow by Language: - English, Search modes - Boolean/Phrase**

Using **AND review of literature or literature review or meta-analysis or systematic review = 205**

**PsycINFO**

MJ breast neoplasms OR breast cancer OR breast n3 (cancer OR neoplasm* OR tumo?r* OR carcinoma) ) OR AB ( breast N3 (cancer OR neoplasm* OR tumor* OR carcinoma

**AND** Antineoplastic agents, Hormonal+ OR Estrogen Receptor Modulators+ OR Estrogen Antagonists+ OR tamoxifen OR anastrozole OR letrozole OR fulvestrant OR exemestane OR toremifene OR raloxifene OR aromatase inhibitor* OR adjuvant endocrine therap* OR adjuvant hormonal therap* OR Antineoplastic agents OR Estrogen Antagonists

**AND** Social support OR Social networks+ OR Social groups OR Social determinants of health OR Social Disadvantage OR Family support OR Family caregivers OR Social support OR Social stigma OR Employment status OR Rural OR Lack of provider caregiver availability OR Poor access to healthcare OR Follow up OR Presence of symptoms OR Disease severity OR Clinical improvement OR Psychiatric condition OR Duration of the disease OR Adverse effects OR Patient friendliness of the regimen OR Perceived effectiveness of the drug OR Duration of the treatment OR Drug type OR Health beliefs OR Knowledge OR Co-morbidities **OR** (adherence OR non adherence OR nonadherence OR compliance OR non compliance OR noncompliance) N3 (treatment OR medication OR patient* ) OR ( persist* or discontinue*) N3 (treatment OR medication OR patient* ) OR ((Treatment of medication) N2 refus*)) OR Treatment Compliance OR Treatment Refusal OR Treatment Dropouts OR Patient Compliance OR Medication compliance" OR Treatment Refusal OR implementation OR discontinuation OR persistence OR initiation OR pharmionics OR adherence OR non adherence OR nonadherence OR compliance OR non compliance OR noncompliance OR (adherence OR non adherence OR non-adherence OR compliance OR non-compliance OR noncompliance) N3 (treatment OR medication OR patient* )

**Expanders - Apply equivalent subjects, Narrow by Methodology: - meta analysis, Narrow by Methodology: - systematic review, Narrow by Methodology: - literature review, Narrow by Language: - English, Search modes - Boolean/Phrase**
