# Supplementary material for: Identifying determinants of adherence to adjuvant endocrine therapy following breast cancer: A systematic review of reviews
Source: Cancer Med. 2024 Jan 19;13(3):e6937. doi: 10.1002/cam4.6937 (PMC10905548; doi:10.1002/cam4.6937)
Supplement: Supplementary file 3 [file CAM4-13-e6937-s004.docx]

**Supplementary File 2: Characteristics of included systematic reviews**

| **Systematic review author & year** | **Review aim/objectives^1^** | **Eligible Data** | **Databases searched & time period** | **Reported restrictions on the search or eligibility** | **Number of included papers** | **Method of synthesis** |
| --- | --- | --- | --- | --- | --- | --- |
| Banning et al., 2012 | Provide a succinct account of existing evidence on adherence with medication in post-menopausal women receiving adjuvant therapy for BC. | Quantitative | PubMed, Medline, Cochrane Library, PsycINFO, British Nursing Index, Advanced Google Scholar (search engine), and hand searching individual journals; 01/1999 - 03/2011 | *Inclusion*: English language only; Discussed adherence with adjuvant therapy in post-menopausal BC patients only.  *Exclusion:* RCT duration <3 months; Failure to address how adherence was measured; Age range unclear; Research search design not accurately discussed. | 13 | Narrative (n=13) |
| Murphy et al., 2012 | Identify correlates of adjuvant ET (SERMs and AIs) adherence and persistence among BC survivors. | Quantitative | Medline, PubMed, PsycINFO, CINAHL, and Scopus (bibliography searching of selected articles only);  1998 - 04/2012 or 05/2012 (end date varied by database). | *Inclusion:* English language only; Peer reviewed primary study only; Female BC survivors prescribed adjuvant ET after completing primary treatment for BC; Correlates of adherence and/or persistence measured or assessed in clinical practice setting.  *Exclusion:* Adherence and/or persistence to extended ET or exclusively after therapy switches; Predominantly male BC population; DCIS; Metastatic disease; No definitive BC diagnosis. | 30^2^ | Narrative (n=29)^3^ |
| Sawesi et al., 2014 | Evaluate the reasons for and factors associated with nonadherence (compliance and persistence) to TAM and AIs among women with BC. | Quantitative or Qualitative | CINAHL, PsycINFO, PubMed, and reference list searching (included studies and relevant reviews); 01/1990 - 10/2011 | *Inclusion:* English language only; Peer reviewed publications only; Reasons for medication non-adherence specified; Medication adherence outcomes reported; Received TAM or AIs; BC diagnosis.  *Exclusion:* Factors other than TAM or AI adherence evaluated (e.g. appointments kept, chemotherapy, radiation). | 26 | Narrative (n=26)^3^ |
| Van Liew et al., 2014 | Evaluate associations between psychosocial factors and BC survivors’ adherence and/or persistence to adjuvant ET. | Quantitative | PsycINFO, PubMed, and reference lists of relevant review papers, and of studies included in the review; All publication years - 01/02/2014 | *Inclusion:* English language only; Peer reviewed full text publications only; Diagnosed at ≥18; Initiated a TAM or AI; ≥1 psychosocial variable; ≥1 ET outcome measure; Quantitative analysis.  *Exclusion:* Adherence promoting interventions; Alternative patient population (e.g. chemoprevention, case study, patients considering but not initiating ET). | 14 | Narrative (n=14)^3^ |
| Cahir et al., 2015 | Identify modifiable determinants of adjuvant ET MTB in women with stage I-III breast cancer in clinical practice settings. | Quantitative | PubMed, Embase, PsycINFO, and CINAHL; Database start – 31/03/2014 | *Inclusion:* Peer reviewed primary study publication only; Female BC patients stages I-III; Prescribed adjuvant ET; Determinants of adjuvant ET MTB measured or assessed in clinical practice settings; Observational study or RCT in clinical practice setting.  *Exclusion:* DCIS; Metastatic disease; ET exclusively used after the initial 5 year period; ET switches; Clinical trials. |  | Narrative (n=45)  Meta-analysis (ORs)   - Treatment side effects: adherence (n=2) & persistence (n=2) - Number of prescription medications: adherence (n=2) & persistence (n=2) - Follow up care with GP vs oncologist: persistence (n=3) |
| Mausbach et al., 2015 | Analyse evidence concerning the effects of depression on non-adherence to AET in women with BC | Quantitative | Medline, PsycINFO, and reviewing references of articles included in the search results for other relevant articles not identified in the original search; Database inception - 01/05/2015 | *Inclusion:* English language only; included discontinuation of non-adherence to, or lack of persistence in taking AET among women with BC as an outcome; assessed depression by semi-structured/structured diagnostic interviews, by ICD-9/-10 diagnostic codes, or by self-report instruments; reported statistical information enabling calculation of an effect size.  *Exclusion*: Assessed adherence to other cancer treatments. | 9 | Meta-analysis (effect sizes; Cohen’s *d*) (n=9) |
| Moon et al., 2017 | Identify predictors of non-adherence and non-persistence to ET in BC survivors. | Quantitative | Medline, Embase, Web of Science,  PsycINFO, CINAHL, grey literature, and reference lists of included articles; Inception – 04/2016 | *Inclusion:* English language only; Full text only; Female; >18 years old; Prescribed adjuvant ET for BC; Study conducted in clinical practice; Present statistical tests of association between ET adherence or persistence and a correlate or predictor.  *Exclusion:* DCIS; Stage IV; Intervention to improve adherence; ET initiation; No primary data. | 61 | Narrative (n=61)^3^ |
| Lambert et al., 2018 | Summarise patient-reported, personal, social, and structural factors that influence BC survivors’ AET adherence and persistence. | Quantitative or  Qualitative | PubMed, Medline, CINAHL, Embase, PsycINFO, and reference lists of relevant primary sources were hand searched; 01/01/1998 – 18/01/2017 | *Inclusion*: English language only; Peer reviewed primary study only; Assessed AET adherence and or persistence through an objective measurement or self report; Statistically significant patient reported factors associated with AET adherence and/or persistence in female BC survivors (quantitative) or included factors women described as influencing AET experience (qualitative).  *Exclusion:* None listed. | 43 | Narrative (n=43)^4^ |
| Paranjpe et al., 2019 | Summarise and identify all patient- and physician-reported barriers associated with non-adherence and non-persistence of oral ET among BC survivors. | Quantitative or Qualitative | PubMed and references of selected articles; 2012-06/2018 | *Inclusion:* English language only; Full text only; Assessment of the barriers associated with non-adherence or non-persistence of oral ET; Patient reported or physician-reported surveys.  *Exclusion:* Study >6 years old; Data-based or gene-based; Systematic reviews; Barriers to oral chemotherapy; Metastatic BC. | 19 | Narrative (n=19)^3^ |
| Xu et al., 2019 | Explore the experience and feelings associated with the AET trajectory in women with BC and what affects their medication-taking behaviour. | Qualitative | PubMed, Embase, CINAHL, PsycINFO, and grey literature; Inception - 28/02/2019 | *Inclusion:* English language only; Full text original publications; Women with BC diagnosis; TAM or AI administered; Interaction between medication and adherence studied (including those factors women state influence their AET experience).  *Exclusion:* Duplicated literature. | 17 | Thematic synthesis, following Thomas & Harden (2008) (n=17) |
| AlOmeir et al., 2020 | Synthesise data from existing qualitative studies to develop an explanatory model of non-adherence and discontinuation with ET. | Qualitative | PubMed, Web of Science, CINAHL, PsycINFO, Wiley Online Library, ProQuest, Google Scholar, Taylor & Francis online, ScienceDirect, SpringerLink, and references of identified articles; 2010-2019 | *Inclusion:* English language only; Primary research; AET adherence in HR-positive BC.  *Exclusion:* Quantitative methods; Reviews; Not BC specific; ET adherence not investigated. | 24 | Meta-synthesis, based on grounded theory (n=24) |
| Clancy et al., 2020 | Synthesise BC patients’ experiences of adherence and persistence to oral ET. | Qualitative | PubMed, Embase, CINAHL, Web of Science, PsycINFO, Proquest, Lenus, Scopus, Rian.ie, EThOS e theses online, DART Europe, and QC check on Google Scholar; Dates not specified; No year limit. | *Inclusion:* English language only; Primary data only; Unpublished theses; Adults ≥18 years old; BC survivors on adjuvant ET (including SERMS and AIs) after completing primary BC treatment; Assessment of patient experiences/perceptions of adherence and persistence to adjuvant ET.  *Exclusion*: None listed. | 24 | Thematic synthesis, following Thomas & Harden (2008) (n=24) |
| Peddie et al., 2021 | Review literature on BC survivors’ lived experiences of ET side-effects and explore how these may be related to non-adherence and non-persistence behaviour. | Qualitative | Cochrane CENTRAL, Medline, Embase, Web of Science, and PsycINFO; Database inception – 05/2020 | *Inclusion:* English language only; Full text primary data study only; Investigated impact of adjuvant ET side effects on adherence and/or persistence; Aged ≥18 years old; Female primary BC survivors; Study conducted in clinical practice or trials; Any publication year.  *Exclusion:* DCIS; Stage IV; ET initiation; Screening or diagnosis studies; Not using human subjects. | 16 | Thematic synthesis, following Thomas & Harden (2008) (n=16) |
| Toivonen et al., 2021 | Identify which potentially modifiable factors are most consistently associated with adherence to AET (including SERMs and AIs). | Quantitative | CINAHL, Ebsco, Embase, Medline, PsycINFO, PubMed, and Web of Science; 1998 – 28/06/2019 | *Inclusion:* English language only; Original peer reviewed data only; Female BC survivor and/or included a small proportion of men (<5%); Any stage; AET with TAM or third generation AIs; Clinical practice setting; Any adherence measure.  *Exclusion:* Women at high risk for cancer and receiving prophylactic ET; Clinical trial examining AET efficacy; Adherence not a primary outcome; AET initiation/non-initiation or medication switching reported; Interventions targeted adherence; Case studies. | 68 | Narrative (n=68) |
| Fleming et al., 2022 | Evaluate and summarise the relationship between ET side-effects and patterns of adherence and persistence. | Quantitative | Cochrane CENTRAL, Medline, Embase, Web of Science, PsycINFO, grey literature databases, and trial registries; Database inception - 03/09/2021 | *Inclusion:* English language only; Full text primary data only; Females aged ≥18 years old; ET prescription for BC; Trial or clinical practice; Statistical tests of association between ET adherence or persistence and side effects as a correlate or predictor.  *Exclusion:* DCIS; Stage 4, ET Interventions to improve adherence reporting no side effects; Screening or diagnosis studies; Non-human subjects. | 62 | Narrative & Harvest Plot (n=62) |
| Montagna et al., 2022 | Analyse factors associated with poor TAM adherence in BC. | Quantitative or Qualitative | Medline; Dates not specified; No year limit. | *Inclusion:* English language only; Published articles.  *Exclusion*: Paediatric trials; Metastatic BC; Review articles; Multiple publications (not the latest version). | 40^5^ | Narrative (n=40)^3,5^ |
| Yussof et al., 2022 | Identify correlates of AET adherence in females with BC. | Quantitative | Medline, Embase, AMED, PsycINFO, International Pharmaceutical Abstracts, APA PsycArticles, reference lists of past systematic reviews, OVID auto alert monitoring alerts, and citation searching; Database inception – 10/2020 or 21/11/2020 (Automated Ovid alerts to 31/7/2021) | *Inclusion:* English language only; Peer reviewed publication; Females; Humans only; Clinical practice; Investigated factors affecting AET adherence; Patient adherence in the implementation or persistence phase measured for a ≥5 years.  *Exclusion:* Adherence exclusively determined from self-report; Other oral anticancer drugs without AET sub analysis; No patient follow up duration specified; Clinical trials; >20% of patients not followed up for full 5 years. | 26 | Narrative (n=26)^3^ |

^1^Aspects of the aims which are relevant to this review of reviews; some of the eligible systematic reviews had multiple aims.

^2^29 studies reported in 30 papers.

^3^Method of/approach to synthesis not reported by authors.

^4^Authors describe as an integrative review.

^5^Number not clearly stated in paper. The cell reports number of studies included in tables, including studies of adherence to TAM and AIs defined by the authors as: studies on adherence to TAM (n=15); studies on adherence to TAM and other ET (n=16)

studies on psycho-social processes underlying TAM non-adherence (n=13).

Abbreviations: BC: Breast cancer; AET: Adjuvant endocrine therapy; AI(s): Aromatase inhibitor(s); AMED: Allied and complementary medicine database; APA: American psychological association; CINAHL: Cumulative index to nursing and allied health literature; DART: Digitial access to research theses; DCIS: Ductal carcinoma in situ; ET: Endocrine therapy; EThOS: e-theses online service; HR: Hormone receptor; ICD: International Classification of Diseases; MTB: Medication taking behaviour; N: Number; ORs: Odds ratios; QC: Quality control; RCT: Randomised controlled trial; TAM: Tamoxifen; TDF: Theoretical domains framework; SERM(s): Selective oestrogen-receptor modulators.
