# Supplementary material for: Identifying determinants of adherence to adjuvant endocrine therapy following breast cancer: A systematic review of reviews
Source: Cancer Med. 2024 Jan 19;13(3):e6937. doi: 10.1002/cam4.6937 (PMC10905548; doi:10.1002/cam4.6937)
Supplement: Supplementary file 4 [file CAM4-13-e6937-s003.docx]

**Supplementary File 3: Quality appraisal for the included systematic reviews**

| **Systematic review author** | **Q1** | **Q2** | **Q3** | **Q4** | **Q5** | **Q6** | **Q7** | **Q8** | **Q9** | **Q10** | **Q11** | **Yes**  **total** |
| --- | --- | --- | --- | --- | --- | --- | --- | --- | --- | --- | --- | --- |
| Banning et al., 2012 | Yes | Unclear | Yes | Yes | Yes | Unclear | Unclear | Yes | No | Yes | Yes | 7 |
| Murphy et al., 2012 | Yes | Yes | Yes | Yes | Yes | Unclear | Unclear | Yes | Unclear | Unclear | Yes | 7 |
| Sawesi et al., 2014 | Yes | Yes | Yes | Yes | No | No | No | Unclear | No | Yes | Yes | 6 |
| Van Liew et al., 2014 | Yes | Yes | Yes | No | No | No | Unclear | Yes | Unclear | Unclear | Yes | 5 |
| Cahir et al., 2015 | Yes | Yes | Yes | Yes | Yes | Yes | Yes | Yes | Unclear | Yes | Yes | 10 |
| Mausbach et al., 2015 | Yes | Yes | Yes | Yes | No | No | No | Yes | Yes | Unclear | Yes | 7 |
| Moon et al., 2017 | Yes | Yes | Yes | Yes | Yes | Yes | Yes | Yes | Unclear | Unclear | Yes | 9 |
| Lambert et al., 2018 | Yes | Yes | Yes | Yes | Yes | Unclear | Yes | Yes | No | Yes | Yes | 9 |
| Paranjpe et al., 2019 | Yes | Yes | No | No | No | No | No | Unclear | No | Yes | Yes | 4 |
| Xu et al.,  2019 | Yes | Yes | Yes | Yes | Yes | Yes | Yes | Yes | Unclear | Yes | Yes | 10 |
| AlOmeir et al., 2020 | Yes | Yes | Yes | Yes | Yes | Yes | Unclear | Yes | Unclear | Yes | Unclear | 8 |
| Clancy et al., 2020 | Yes | Yes | Yes | Yes | Yes | Yes | No | Yes | Unclear | Yes | NA | 8 |
| Peddie et al., 2021 | Yes | Yes | Yes | Yes | Yes | Yes | Yes | Yes | Unclear | Yes | Yes | 10 |
| Toivonen et al., 2021 | Yes | Yes | Yes | Yes | Yes | Unclear | Yes | Yes | No | Unclear | Yes | 8 |
| Fleming et al., 2022 | Unclear | Yes | Yes | Yes | Yes | Yes | Yes | Yes | Yes | N/A | Yes | 9 |
| Montagna et al., 2022 | Yes | Yes | No | No | No | No | No | No | No | Yes | No | 3 |
| Yussof et al., 2022 | Yes | Yes | Yes | Yes | Yes | Unclear | Yes | Yes | Yes | Yes | Yes | 10 |

Q1, Is the review question clearly stated?; Q2, Were the inclusion criteria for the review appropriate?; Q3, Was the search strategy appropriate?; Q4, Were the sources and resources used to search for studies adequate?; Q5, Were the criteria for appraising studies appropriate?; Q6, Was critical appraisal conducted by two or more reviewers independently?; Q7, Were there methods to minimise errors in data extraction?; Q8 Were the methods used to combine studies appropriate?; Q9, Was the likelihood of publication bias assessed?; Q10, Were recommendations for policy and/or practice supported by the reported data?; Q11, Were the specific directives for new research appropriate?
